# COVID-19 outbreak and its monetary implications for dental practices, hospitals and healthcare workers

**DOI:** 10.1136/postgradmedj-2020-137781

**Published:** 2020-04-03

**Authors:** Imran Farooq, Saqib Ali

**Affiliations:** Department of Biomedical Dental Sciences, College of Dentistry, Imam Abdulrahman Bin Faisal University, Dammam, Saudi Arabia; Department of Biomedical Dental Sciences, College of Dentistry, Imam Abdulrahman Bin Faisal University, Dammam, Saudi Arabia

**Keywords:** infectious diseases

## Abstract

The novel COVID-19 came under limelight few months back (December 2019) and has recently been declared a pandemic by WHO. It has resulted in serious financial implications being faced by dental practices, hospitals and healthcare workers. Dental practice currently is restricted to provision of emergency dental care whereas, many hospitals have also cancelled elective procedures to save finances for COVID-19 treatment which is expensive and unpredictable. In addition, healthcare workers are also facing financial challenges in this difficult time. Competent authorities should step in to help dental practices, hospitals and healthcare workers in order to ensure the provision of all types of healthcare efficiently in these testing times and beyond.

The novel COVID-19 came under limelight few months back (December 2019) and has recently been declared a pandemic by WHO.^1^ The COVID-19 is believed to be transferred from animals to humans.^2^ The other two similar viruses discovered earlier were severe acute respiratory syndrome coronavirus (SARS-COV) and Middle east respiratory syndrome.^3^ The COVID-19 has also been called SARS-CoV-2 but the former name has gained more popularity than the latter name.^4^ The COVID-19 causes human illnesses which could range from a common cold to more severe respiratory problems like pneumonia.^5^ Unfortunately, there is no vaccine available for COVID-19 and the only form of treatment available is to provide symptomatic treatment to the patients with oxygen supplementation.^6^

Dentistry is a profession where the dentist works in close proximity to the patient’s mouth and many procedures produce aerosol which is a mixture of water (from a dental instrument like high speed hand piece) and patient’s saliva or blood.^7^ These aerosols could result in the spread of infection and diseases including COVID-19.^8^ In a recent article published by the *New York Times* has identified dentists who are at the highest risk of getting COVID-19 from their patients due to cross infection.^9^ Due to this, many health regulatory bodies globally (National Health Commission of China, American Dental Association, Consejo General of Spain and National Health Service, UK) have advised their registered dental practitioners to perform only emergency dental procedures and avoid all elective treatments like restoration and extraction of asymptomatic teeth, aesthetic dental procedures, orthodontic adjustments and routine radiographs.^10–13^

This step should be appreciated as it limits the spread of COVID-19; however, it has resulted in serious monetary implications for dental practices worldwide. General dental practices are currently suffering huge financial losses as they can provide only emergency dental care. In a recent survey conducted by Irish Dental Association (IDA) which involved 369 dentists, it was reported that one-fifth of the dentists have closed their practices (temporarily or permanently).^14^ In addition, around three-fourth of the participants are expecting a financial loss of over 70% amid COVID-19 outbreak.^14^ The British Dental Association has also indicated the same and it is expected that dental practices in the UK are going to face crippling losses due to suspension of routine dental care.^15^ In the USA, some practices are closed whereas, some practice owners are not closing it due to the fear of economic losses and non-payment of salaries to their employees.^16^ Reports from other countries have not emerged yet but it is expected that whenever they are published, they are also going to report huge monetary losses incurred by dental practices during the COVID-19 outbreak period.

The governments and dental regulatory bodies of many high-income countries have understood the gravity of the situation and have offered support to dental practices. The Canadian government has set up an Economic Response Plan on 18 March 2020 to support businesses and has kept $C27 billion to support businesses.^17^ The dentists can also apply for this support and save their practices from immediate closure due to financial losses. The government of the UK is also inviting business owners to contact them for loans or credit if they are facing issues regarding payment of salaries or supplies.^18^ The dental practices which come under the umbrella of NHS in the UK, are going to receive some funds to reimburse losses due to COVID-19 outbreak.^15^ The IDA in Ireland has clearly mentioned on their website that Irish government is going to support businesses including dental practices which have been affected by COVID-19 outbreak and is ready to give COVID-19 Business Loan ranging between €5000 and €50 000.^19  20^

While this is the situation in high-income countries, no visible policy to support dental practices in this difficult time has been put forward by the governments or dental regulatory bodies of low-income and middle-income countries. It is highly recommended that competent authorities from these countries should step up and support dental practices which are on the brink of closure due to financial losses given to them by COVID-19 pandemic.

Concerning financial situation of hospitals, the authors believe that their situation is not so different to dental practices as well. Medical doctors and support staff are on the frontline of every medical situation and postemergence of COVID-19, their role has become more substantial.^21^ Unfortunately, the outbreak of COVID-19 has resulted in serious financial pressures being put on hospitals and healthcare workers as they fight this pandemic. Many hospitals in the USA have also cancelled elective procedures and are trying to preserve their finances for the treatment of patients with COVID-19.^22^ The treatment of COVID-19 is expensive and unpredictable and requires a good source of backup finances.^22^

In the USA, a letter has been sent to Speaker of House (Nanci Pelosi) from American Hospital Association President and CEO, American Medical Association Executive Vice President and CEO and American Nurses Association Enterprise CEO asking the Congress to urgently provide US$100 billion funding directly to hospitals and healthcare workers.^23^ The letter indicates the diminishing supplies of personal protective equipment like gowns, masks, N95 respirators, eye protection and diagnostic kits.^23^ In addition, the letter also states that due to long working hours and school closures, medical personnel need to access childcare support and require immediate funding.^23^ This is concerning the staff who are directly dealing with patients with COVID-19. On the other hand, difficulties faced by medical staff who are not directly involved in this situation are significant as well. St. Claire HealthCare in Morehead, Kentucky, USA has recently issued a statement in which they have mentioned that they are laying off quarter of their staff temporarily who are not directly involved in the care of patients with COVID-19.^24^

This situation is alarming and requires urgent financial support that should be offered to hospitals and all healthcare workers to counter the losses sustained and to meet their urgent needs.
